# A Resolution to the Blue Whiting (*Micromesistius poutassou*) Population Paradox?

**DOI:** 10.1371/journal.pone.0106237

**Published:** 2014-09-03

**Authors:** Fabien Pointin, Mark R. Payne

**Affiliations:** Centre for Ocean Life, National Institute of Aquatic Resources (DTU-Aqua), Technical University of Denmark, Charlottenlund, Denmark; Aristotle University of Thessaloniki, Greece

## Abstract

We provide the strongest evidence to date supporting the existence of two independent blue whiting (*Micromesistius poutassou* (Risso, 1827)) populations in the North Atlantic. In spite of extensive data collected in conjunction with the fishery, the population structure of blue whiting is poorly understood. On one hand, genetic, morphometric, otolith and drift modelling studies point towards the existence of two populations, but, on the other hand, observations of adult distributions point towards a single population. A paradox therefore arises in attempting to reconcile these two sets of information. Here we analyse 1100 observations of blue whiting larvae from the Continuous Plankton Recorder (CPR) from 1948–2005 using modern statistical techniques. We show a clear spatial separation between a northern spawning area, in the Rockall Trough, and a southern one, off the Porcupine Seabight. We further show a difference in the timing of spawning between these sites of at least a month, and meaningful differences in interannual variability. The results therefore support the two-population hypothesis. Furthermore, we resolve the paradox by showing that the acoustic observations cited in support of the single-population model are not capable of resolving both populations, as they occur too late in the year and do not extend sufficiently far south to cover the southern population: the confusion is the result of a simple observational artefact. We conclude that blue whiting in the North Atlantic comprises two populations.

## Introduction

Blue whiting (*Micromesistius poutassou* (Risso, 1827)) is a small mesopelagic planktivorous gadoid found throughout the North-East Atlantic. The species has been the subject of a large but highly variable commercial fishery since the late 1970s. Fisheries surveys and formal stock assessments have been in place since the early 1980s, and management agreements in more recent times. The first scientific reports date back more than a century [1] and the species is generally regarded as playing an important role in the ecology of the North-East Atlantic [2].

In recent decades the stock (and the associated fishery) has undergone dramatic changes. From moderate levels in the early 1990s, the stock and fishery swelled during the late 1990s and early 2000s: in 2004, landings reached 2.4 millions tonnes, making it the third largest marine fishery in the world [3]. The stock has since reduced dramatically in size [4], however, and at one point, scientific advice recommended the closure of the fishery altogether [5]. The most recent stock assessments suggest that the decline has stabilised and that the population may be increasing again [6]. Yet, in spite of the relative importance of this fish population, and the wealth of information and studies that normally are associated with an assessed species, there are still important gaps in our understanding.

**Figure 1:**
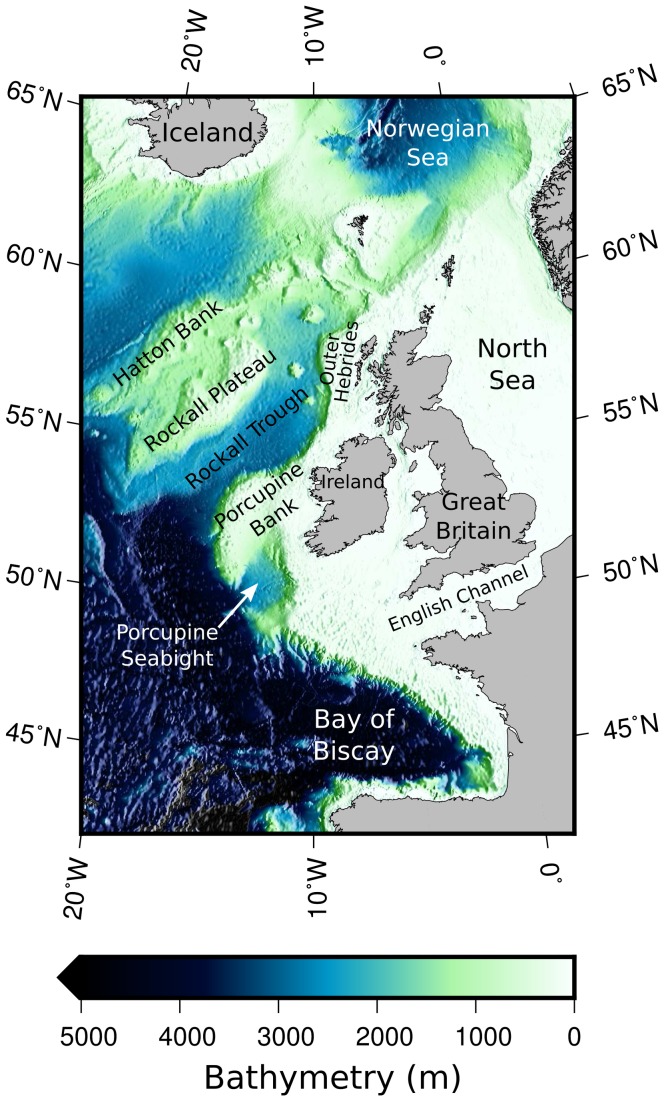
Bathymetric relief map of the study area. Features mentioned in the text are labelled.

One such outstanding question is that of population structure. The species is widely distributed throughout the North-East Atlantic. The core of the distributional range is from the Bay of Biscay along the continental shelf edge to the Norwegian Sea (Figure 1). The edges of the distribution include the southern Iberian Peninsula and the Mediterranean Sea, the Barents Sea, the North Sea (although not the Baltic) and the Mid-Atlantic ridge, East-Greenland and the east coast of North America [7], [8]. The Mediterranean population is typically considered as a separate population that is isolated from the rest of the Atlantic population and is not considered further here.

However, the Atlantic population structure, if any, is the subject of some controversy. One long-running line of argument (see *e.g.* [7] for early references) proposes the existence of two separate Atlantic populations. According to this hypothesis, one population (hereafter the northerly population) spawns in spring to the west of Great Britain and the Outer Hebrides along the continental shelf edge, in the Rockall Trough and around the Rockall Plateau and Hatton Bank: this population then migrates northwards into the Norwegian and Barents Seas where it feeds during summer, and possibly overwinters. The second (southerly) population is thought to spawn around Porcupine Bank and the Porcupine Seabight, and possibly further to the south in the Bay of Biscay. This population may migrate southwards to the Bay of Biscay to feed during summer, although the understanding of the migrations and distributions in this region is limited.

A variety of different studies support this hypothesis. Early genetic studies based on allozyme markers were able to show differences between individuals caught at the edges of the distribution [9], [10] (*e.g.* between the Mediterranean and Barents Seas). More modern studies based on microsatellite loci [11], [12] have provided more detail, with differences exhibited between individuals from the Hebrides and Porcupine Bank. Growth studies based on the larval region of otoliths captured from adults suggested that individuals captured in southern areas (Porcupine Bank and Bay of Biscay) grew significantly faster during their larval stage than those from northern areas (the Hebrides and Norwegian sea), suggesting that fish from these regions do not mix randomly [13]. Otolith shape analysis [14] suggests systematic differences between the Celtic Sea and the Norwegian Sea. Morphometric and meristic data also support a separation between the Hebrides and Porcupine Bank [15]. Circulation studies lend further support to this idea by providing a mechanism that can maintain the separation: larvae spawned north of 53–55 °N are advected northwards, while those south of this region drift southwards [16]–[18].

The current management structure, however, does not reflect this evidence. Blue whiting in the North-East Atlantic is managed as a single stock, with one quota to cover the entire domain. This was not always the case: the initial management structure upon establishment of the ICES Blue Whiting Assessment Working Group in the early 1980s was a two-population construct. Surveys performed during this time were often reported in terms of southern and northern populations, and separate abundance estimates were generated for each population (*e.g.* [19]). However, the two populations were merged into a single stock in 1993, due to reasons of convenience and the absence of data to the contrary [18].

During the intervening two decades, the single-stock paradigm has come to dominate both the management of this stock and the science performed upon it. Most modern publications on this topic (*e.g.* [4], [20]–[22]) start from this assumption and interpret their results in terms of a single population. Recent management advice even goes so far as to deny any evidence to the contrary, stating “…*there is no scientific evidence in support of multiple stocks with distinct spawning locations or timings.*” [6]. On the other hand, the steady accumulation of results undermining the single-stock paradigm has lead to blue whiting being cited as an example of the mismatch between genetic studies and management [23].

**Figure 2:**
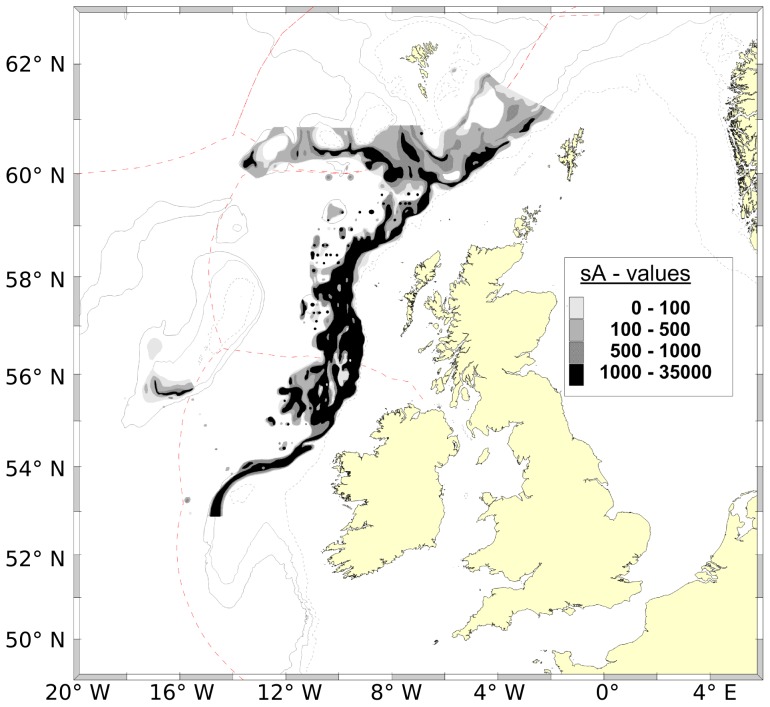
Distribution of the blue whiting spawning stock from a fisheries acoustic survey. The acoustic intensity of blue whiting (sA, which is directly related to abundance) from the International Blue Whiting Spawning Stock Survey (IBWSS) is shown for the 2013 acoustic survey [52]. Isobaths are plotted as grey lines. International maritime boundaries are plotted as red dotted lines. Note the continuous distribution along the shelf edge and limited southern extension of the survey.

Part of the reason for the dominance of the single-stock approach lies in the observations of blue whiting on the spawning grounds. Acoustic fisheries surveys have covered the spawning grounds since the early 1980s, and are generally regarded as one of the best sources of information about the spatial distribution of this species. Such surveys, however, generally show a continuum of fish running from the Hebrides all the way to Porcupine Bank (Figure 2). The question can therefore be raised: if, as the two-population hypothesis suggests, there are truly two populations with separate spawning grounds, why can we not see them in the surveys? Alternatively, if, as the acoustic observations suggest, there is mixing at spawning time, how can the genetic and morphometric separations observed be maintained? It is this paradox, with a conflict between two conceptual models, both of which seem reasonable when viewed individually but are nevertheless mutually exclusive, that is at the core of the conflict between the two models of blue whiting population structure.

Resolving this controversy requires a fresh approach. One potential data source that could shed new light on this issue is the Continuous Plankton Recorder (CPR). The CPR is a sampling device that is towed behind ships of opportunity throughout European waters (and more recently on a global scale) and captures both phytoplankton and zooplankton together with fish eggs and larvae [24]. Starting in 1931, it is one of the longest running biological sampling programs in the world, and provides a unique and invaluable insight into the dynamics of marine systems. The CPR is especially closely linked to the history of blue whiting: the species is one of the most commonly occurring fish species in the CPR record, comprising approximately 10% of all fish ichthyoplankton identified [25] and 75% of all larvae west of the British Isles [26]. The broad spatial and temporal coverage of the CPR, and its penchant for blue whiting, lead to the identification of large concentrations of blue whiting larvae around Rockall Trough and Rockall Plateau in the 1950s [27], [28], and the CPR is therefore frequently credited as playing a crucial role in the identification and development of the fishery [29]. The same broad coverage can potentially shed fresh light on the population structure of this species.

In this work, we aim to investigate the population structure of blue whiting using the CPR larval observations. In particular, we will apply modern statistical modelling techniques to this unique dataset to develop a comprehensive overview of the spatial and temporal distribution of the spawning products. These results can then be used to assess support for the various conceptual models of blue whiting population structure in the North-East Atlantic.

## Materials and Methods

### Continuous Plankton Recorder (CPR) Data

The CPR is towed behind ships of opportunity at depths of 7 m to 10 m. Sea water enters the recorder through a small opening in the front of the device, and is filtered through a silk screen with a mesh size of approximately 270 *µ*m. The silk cloth is stored on a roll and replaced continuously as the recorder is towed through the water: after being exposed to sea water, the cloth is covered with a second layer of unexposed silk and then enters a tank of formalin to preserve the samples. On shore, the silk is divided into squares that correspond to approximately 10 nautical miles of towing distance, and analysed under a microscope by a taxonomist. Details of the sampling and analysis procedure are published elsewhere [24].

Initially, all fish larvae were identified to species level on all samples. Reductions in funding in the late 1970s lead to the cessation of species-level identification from the early 1980s onwards: fish larvae after this point were noted but not identified. However, a new initiative was commenced in the late 2000s and with funding from the UK government the archived fish larvae were reanalysed to species level in a restricted region around the British Isles [30].

CPR blue whiting larval observations were provided upon request by the Sir Alister Hardy Foundation for Ocean Science (SAHFOS), Plymouth, UK. In addition to the spatial domain incorporated in the modern reanalysis project (from 20 °W to 10 °E and 44 °N to 64 °N), we also obtained observations back to 1948 over the entire North Atlantic domain. Both presence and absence observations were incorporated in the data obtained.

### Modelling approach

The goal of our data analysis was to find a model that synthesizes the data available and accounts for the complex spatial-temporal distribution of the samples. We apply an Information Theoretic approach to the development of this model [31], defining an ensemble of candidate model structures in advance and fitting them to the observations. We then choose the model that gives the most parsimonious representation of the data, as judged by the Akaike Information Criteria (AIC), a metric that balances the fit to the data against the complexity of the model (number of parameters employed). The “best” model is the one with the lowest AIC score.

We differ from previous analyses of CPR fish larval data (*e.g.* [30], [32], [33]) by disregarding abundance data. CPR fish data are not recorded as true abundances, but rather as abundance categories. Beyond the first categories (0, 1, 2, and 3 larvae), where there is an unambiguous relationship between the number of larvae and the category, there is a rapid loss of information *e.g.* the next categories are 4–11, and 12–25. The approaches applied by other authors, typically assuming a Gaussian or Poisson observation model, are therefore not valid in this case. A statistically valid model to handle this observational structure would require a high degree of sophistication, based, for example, on continuation ratio logits [34]. We choose instead to simplify the problem by disregarding the abundance information and instead focusing on the presence/absence aspect of the data.

Considering the CPR data as presence/absence observations lends itself naturally to Generalised Additive Models (GAMs) with a Bernoulli observational structure. We employ a GAM using the metadata of each observation (spatial position, year, day of year and time of day) as the basis for the explanatory variables. Specifically, we employ the following model structure:
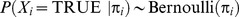
(1a)

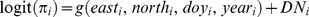
(1b)where 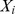 is presence/absence observation 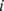, 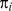 is the probability of 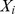 being true (present), and 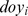, and 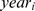 are the day of year and year of the observation. The spatial domain is represented in the Universal Transverse Mercator (UTM) projection (Zone 28) to minimise the effect of coordinate distortions due to the curvature of the earth. The spatial position is thus represented by the eastings, 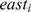, and northings, 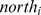, in Equation 1b above.

The variable 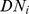 is a categorical factor indicating whether the sample was taken during the day or night. The ability of the CPR to capture fish larvae may change with the light environment, due to either active avoidance of the gear or diel vertical migrations of the larvae. The 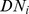 variable was therefore incorporated to account for such effects and was based on the solar-elevation at the time and position of each observation, as calculated using the solarpos() function in the “maptools” package in R [35]. Sunrise/sunset were defined following the “civil dawn” convention *i.e.* night is where the sun is six degrees or more below the horizon. The 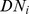 term was used in all models considered.

The function 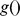 in Equation 1b is the main unknown element. We consider an ensemble of different terms for 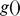, ranging from a fully separable model, where each space-time dimension influences the probability of occurrence independently, to full three-dimensional interactions between space and day of year. We do not consider four dimensional interactions (*i.e.* space - day-of-year - year interactions), due to the limited number of presence observations.

Two different structures are considered for the year term. The first, and simplest model does not consider a year term, and simply assumes the abundance of larvae in each year to be the same. Alternatively, interannual variations in adult abundance (and therefore of the probability of observing larvae) were accounted for as smoothly varying covariates of time (denoted by an 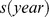 term in 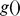).

The full list of models considered is given in Table 1.

**Table 1:** Model fitting results.

Model	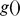 function	Comps.	Dev. Expl.	AUC	AIC	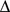 AIC 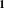	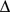 AIC 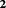	DN
1	east + north + doy	1	0.384	0.945	6930	1127	1199	0.20
2	east + north + doy + syear	1	0.424	0.953	6558	755	827	0.18
3	east*north + doy	1	0.388	0.947	6918	1116	1187	0.21
4	east*north + doy + syear	1	0.432	0.956	6515	712	784	0.22
5	east*north*doy	1	0.465	0.966	6127	324	396	0.18
6	east*north*doy + syear	1	0.501	0.970	5803	0	72	0.18
7	east*north + doy + syear 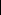 comp	2	0.445	0.959	6396		665	0.22
8	east*north + doy 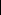 comp + syear	2	0.464	0.964	6165		434	0.20
9	east*north + doy 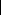 comp + syear 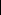 comp	2	0.471	0.964	6108		377	0.20
10	east*north*doy + syear 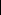 comp	2	0.510	0.972	5731		0	0.17

The 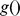 function is the representation of space and time in the model: “*” indicates the use of 2D or 3D interaction smoothers, whereas “+” indicates additive terms. “
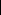
comp” indicates that the term is conditional on the component (northern or southern component). “syear” indicates a spline smoother year-term. Comps: The number of components fitted in the model. AIC: Aikaike Information Criteria. AUC: Area under the Curve (of a Receiver Operator Curve). Dev. Expl..: the fraction of deviance explained by the model. 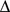
AIC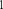: Difference in the AIC from the minimum AIC of the first model ensemble 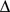
AIC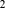: Difference in the AIC from the minimum AIC of the second model ensemble (multicomponent models). 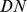: The value of the day-night coefficient in the model.

Models were fitted using the mgcv package in R [36], [37]. Following the recommendation of [36], each model is fitted with a “gamma” parameter set to 1.4, to avoid overfitting. Cyclic cubic regression splines were used as smoothers for day of year: standard cubic regression splines were used for all other terms. Two and three dimensional tensor-product interaction smoothers [36], [38] were used for interaction terms, where appropriate.

### Model validation and evaluation

Model validation for models with non-Gaussian responses is somewhat more challenging than for standard linear modelling, where an array of diagnostic plots exist to assess the validity of the fit. This is particularly the case for a binary response variable, such as the presence/absence observations used here, where the concept of a residual becomes difficult to interpret. Binary response variables are, by definition, Bernoulli distributed, so there are no distributional assumptions to check.

Our model validation is therefore limited to checking that the smoothers are neither over-constrained nor are overfitting. We follow the guidelines described in [36] and in the internal documentation of the mgcv package in this regard, relying heavily on the gam.check() function.

We assess model goodness of fit using two standard measures. The area-under-the-curve (AUC) of a receiver-operator curve (ROC) is a commonly employed measure of the ability of a model to distinguish between binary outcomes. A value of 1 indicates perfect discrimination between presence and absence, whilst a value of 0.5 is that expected from a random number generator: models with values in excess of 0.75 are typically regarded as having a “useful” ability to discriminate between absences and presences [39], [40]. Although the validity of this metric has been questioned [41], we present these results here for consistency with other analyses. The AUC for each model was calculated using the verification package in R [42]. We also considered the “explained deviance” as a second metric of the model goodness of fit [36]: this metric can be considered as an analogue of the coefficient of determination, *R* ^2^, for generalised linear and generalised additive models.

Model fits were visualised by evaluating the fitted model on a regular three dimensional grid (*east*, *north*, *doy*) for a given year. The annual distributions were then normalised and the mean marginal distributions determined. Interannual variability in spawning was visualised by integrating the probability of larval occurrence across these grids for each year, with confidence intervals generated by resampling from the posterior distribution of the fit [36].

## Results

### Data exploration

**Figure 3:**
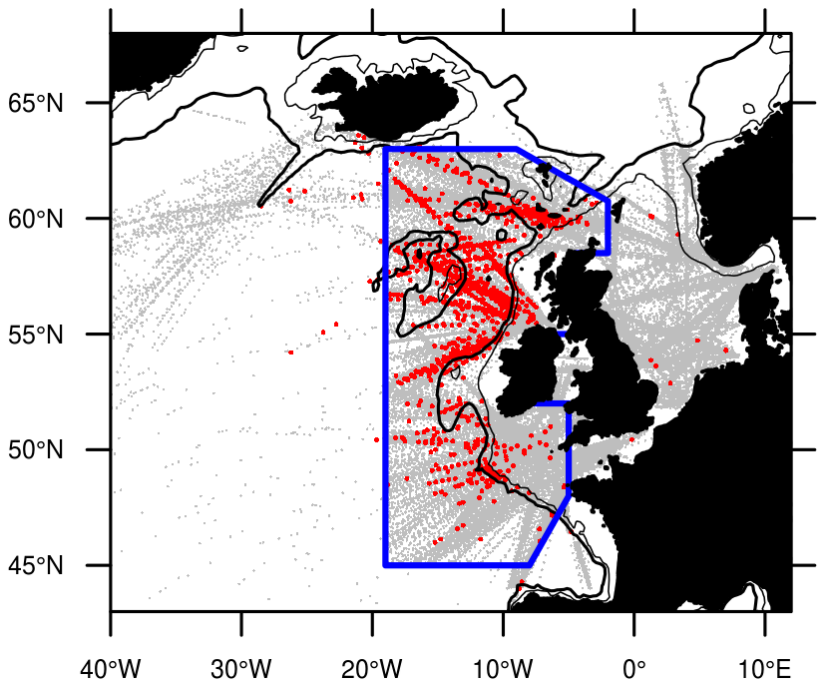
Spatial distribution of CPR samples. Grey points are locations where CPR samples have been checked for fish larvae. Red circles are where these samples were found to contain blue whiting. The blue box denotes the spatial region of interest used in further model-based analyses.

In total, 134 260 CPR observations that had been checked for blue whiting larvae were obtained in the North Atlantic region. The spatial distribution of these samples clearly shows a high concentration of samples in the North Sea and to the west of Great Britain and Ireland, from the continental shelf out to approximately 20°W, north of the Iberian peninsula, and south of Iceland (Figure 3). Discontinuities and inhomogeneities arise in the spatial distribution of samples due to both the pattern of shipping routes employed by the CPR, and the boundaries imposed by the modern reanalysis project (which is focused on the North Sea, and the waters to the west of Great Britain and Ireland).

The domain covered by the modern reanalysis is, fortunately, also the region that clearly contains the most blue whiting larval observations. A few presences are seen outside of this region, particularly towards the Mid-Atlantic ridge, and are consistent with other reports [43]. However, the presence of blue whiting larvae in the North Sea and English Channel has not been reported previously, and is not consistent with existing knowledge. We have therefore interpreted these observations as misidentifications or errors in data entry.

In order to simplify the analysis, we focus the modelling efforts on the region of highest sampling density and most frequent larval-presence, as denoted by the region in Figure 3. The region-of-interest polygon is drawn to follow the boundaries of the modern reanalysis to the west of Great Britain and Ireland. Regions in the Norwegian Sea and Bay of Biscay are also excluded, due to sparse sampling coverage. 34 out of 1161 presence observations are excluded by this spatial filtering, an acceptably low number (3%) that highlights the peripheral nature of these regions. The final data set consisted of 59 042 observations, of which 1127 were presences (1.9%).

**Figure 4:**
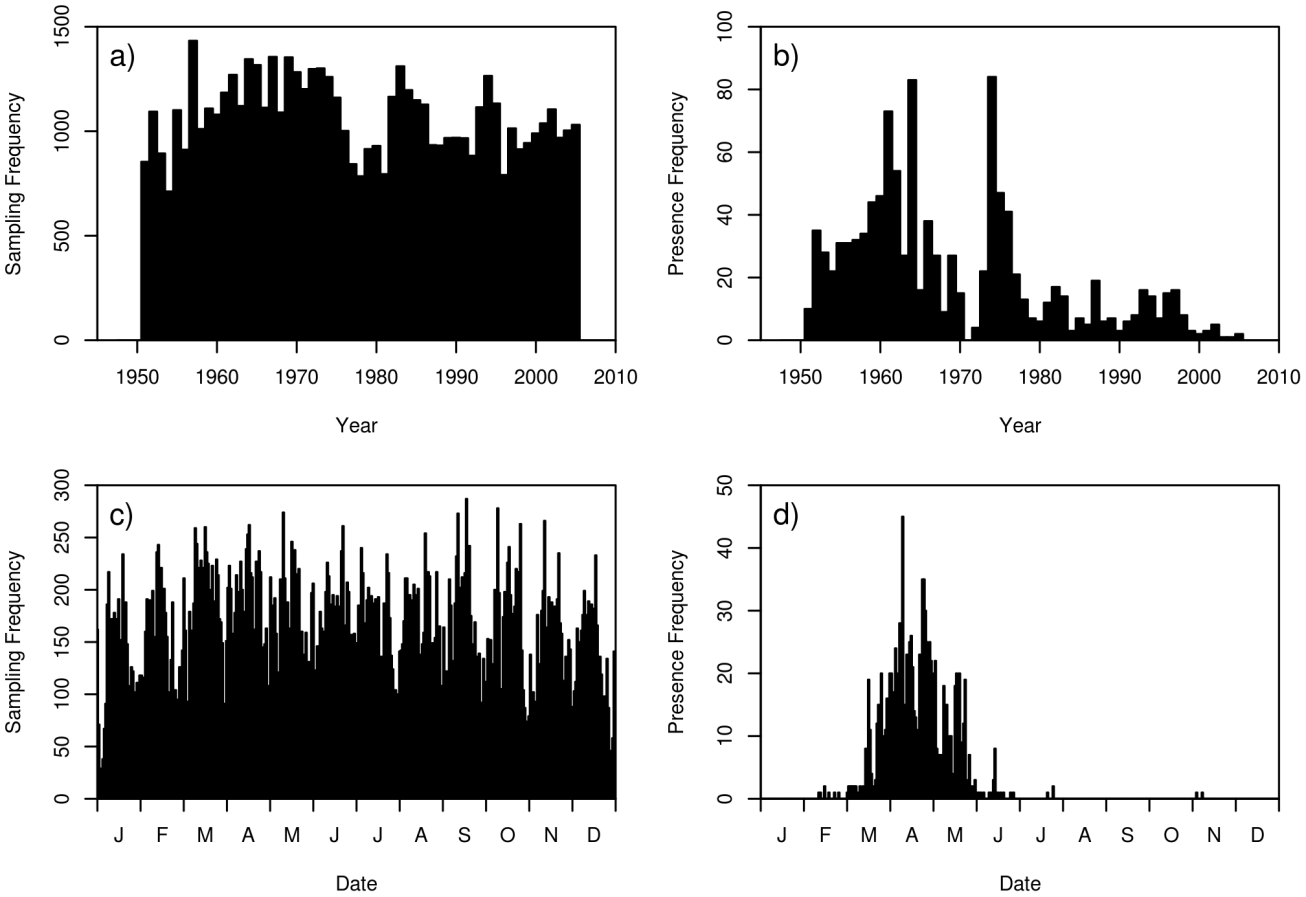
Temporal distribution of CPR samples. Temporal distribution of samples checked for blue whiting larvae obtained from the CPR in the region of interest outlined in Figure 3. a) Sampling frequency in each year b) Presence frequency in each year c) Sampling frequency as a function of date in the year d) Presence frequency as a function of date in the year. In a) and b), each bar corresponds to a single year, whilst in c) and d) it corresponds to a day of year.

The interannual distribution of the samples and the presences in the study region show a number of systematic patterns (Figure 4). Although the annual distribution of samples is relatively constant (Figure 4a), the number of presences reported varies over time (Figure 4b), and is markedly reduced from 1975 onwards. This reduction can be explained in part by a closer examination of the spatial distribution of samples in each year (Figure S1). Sampling intensity in the Rockall region in particular was reduced during this time and is associated with the close of the ocean weather ships in this region (and their associated CPR routes) and may account for the changes in the frequency of presence observations.

The distribution of samples with respect to the day of year immediately reveals the spawning period of blue whiting. The CPR samples are uniformly spread throughout the year, although there is a clear monthly sampling cycle, with the greatest sampling intensity in the middle of each month (Figure 4c). However blue whiting larvae are predominately found in the months of March, April and May, with two outliers occurring in November (Figure 4d). These observations may be erroneous but in the absence of other information, are retained in the analysis.

**Figure 5:**
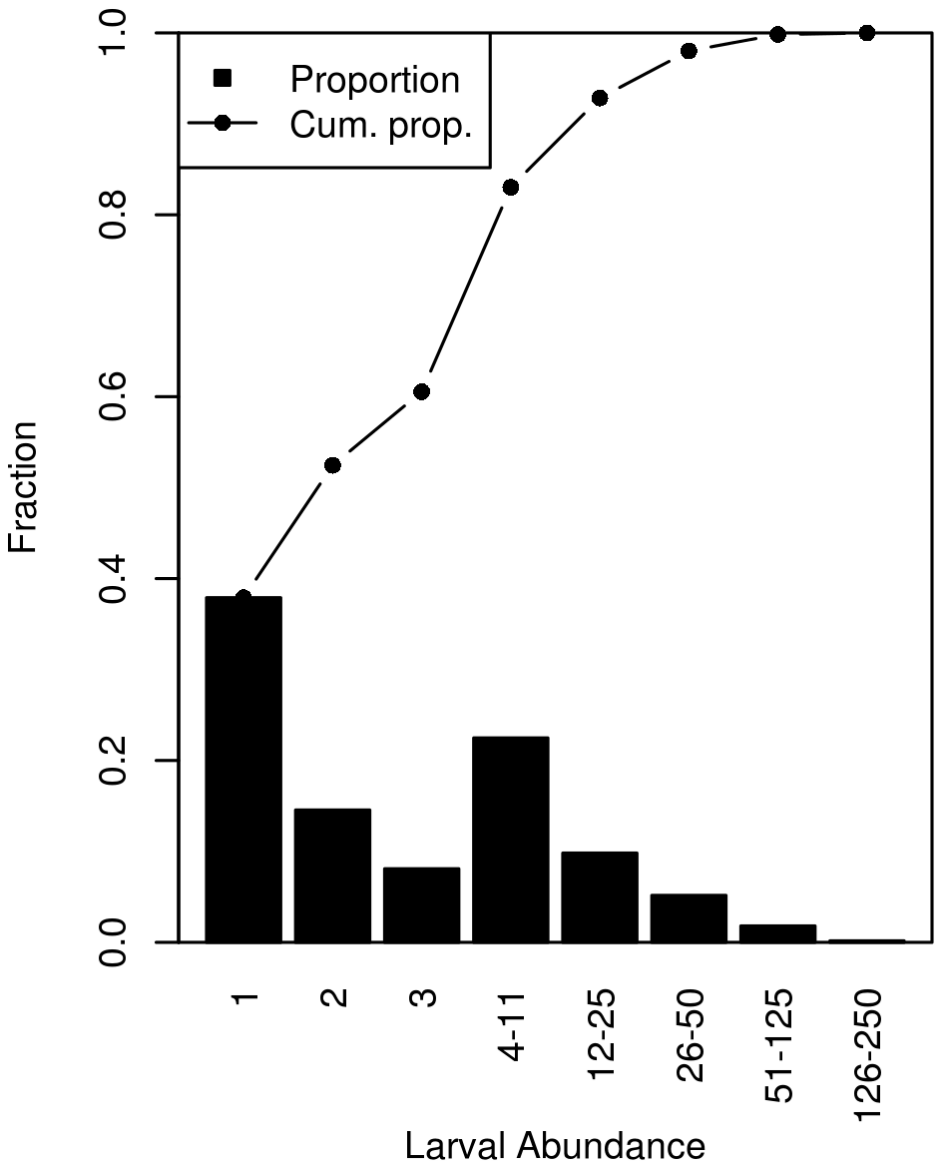
Distribution of larval abundances reported in the CPR. The relative proportion of each non-zero abundance category reported (bars) and the cumulative proportion (line) are show. Cumulative proportion is defined here as the proportion of presences with an abundance less than or equal to the given category. Note that the abundances are the abundance categories reported by the CPR survey [24]. Observations of zero larvae (absence) are omitted from this distribution.

The distribution of larval abundances supports the choice of presence/absence modelling (Figure 5). Of the approximately 1100 presence observations, 60% are of abundance category 1, 2 or 3, and can therefore be directly related to their actual abundance. However, the remaining 40% are reported as abundance ranges which are not readily modelled using standard statistical techniques. Based on these results, the decision to employ presence/absence modelling appears justified.

### Model fitting and validation

The quality of the fits from the initial model ensemble (Models 1–6 in Table 1) showed a strong dependence on the space-time formulation, 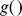, employed. Increasing the degree of interaction between space and time increased the quality of the model fit to a degree that outweighed the penalties associated with the addition of extra fitting parameters (as judged by the AIC criteria). The quality of the fit also improved, as judged by both the deviance explained and the area under the receiver-operator curve (AUC) statistics. Models that were fully separable, with no interaction terms were the worst, whilst those with full three-dimensional interactions between eastings, northings and day of year were the best according to both of these criteria. Year effects were clearly required. In each of the three cases, for a constant space and day-of-year formulation, adding the year effect lead to a better quality model. Model 6 is clearly the best of these candidate models, with the next best model (Model 5) having an AIC value more than 300 units greater: 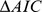 values of more than 20 are typically characterised as a model having “essentially no empirical support” [31], [44].

However, initial model evaluation suggested a further refinement to the model ensemble that had hereto been overlooked. All models showed a clear local minimum in the density of blue whiting larvae on Porcupine Bank (Figure S2), between approximately 52 and 54°N, with spawning centres to the north and south of this feature. This result is clearly in line with other published results suggesting the presence of two-populations. Furthermore, these two spawning regions also appear to have distinct spawning times that are separated by a month or more (Figure S3). There is thus a clear suggestion of two distinct spawning-grounds in these results.

A second ensemble of models was therefore generated by expanding the first to include this alternative structure. Specifically, we drew a dividing line at 53°N based on the results of Model 6 (see Figures S2 and S3). Larvae observed north of this line are associated with the “northern component”, and those south of the line are associated with the “southern component”. Models allowing for component-dependent interannual abundance variations (Model 7), component-dependent spawning times (Model 8) or both (Models 9–10) were created and fitted.

The two-component models are systematically better than their corresponding single-spawning-ground models (Table 1). The addition of the two-component feature leads to a substantial reduction in the AIC and increase in the AUC in models where there is no interaction between space and season (day of year) (*i.e.* Model 4 compared with model 7). Model 10 which incorporates full space-season interaction with component-dependent interannual variations in abundance, is clearly an improvement on its one-component counterpart (Model 6), and is now the best model overall.

All models appear to fit the data well. Model validity checks performed as part of the fitting procedure suggest that the smoothers are capturing the variability. The models also capture the majority of the deviance (Model 10 captures 51%). The AUC scores are particularly impressive, and exceed 95% for nearly all models, suggesting a high degree of skill in discriminating between the presence and absence of larvae, although may be unrealistically high due to the low number of presences. The model fits therefore appear valid representations of the data, and the best fitting model, Model 10, is therefore adopted as the basis for the remainder of this study.

### Model visualisation

**Figure 6:**
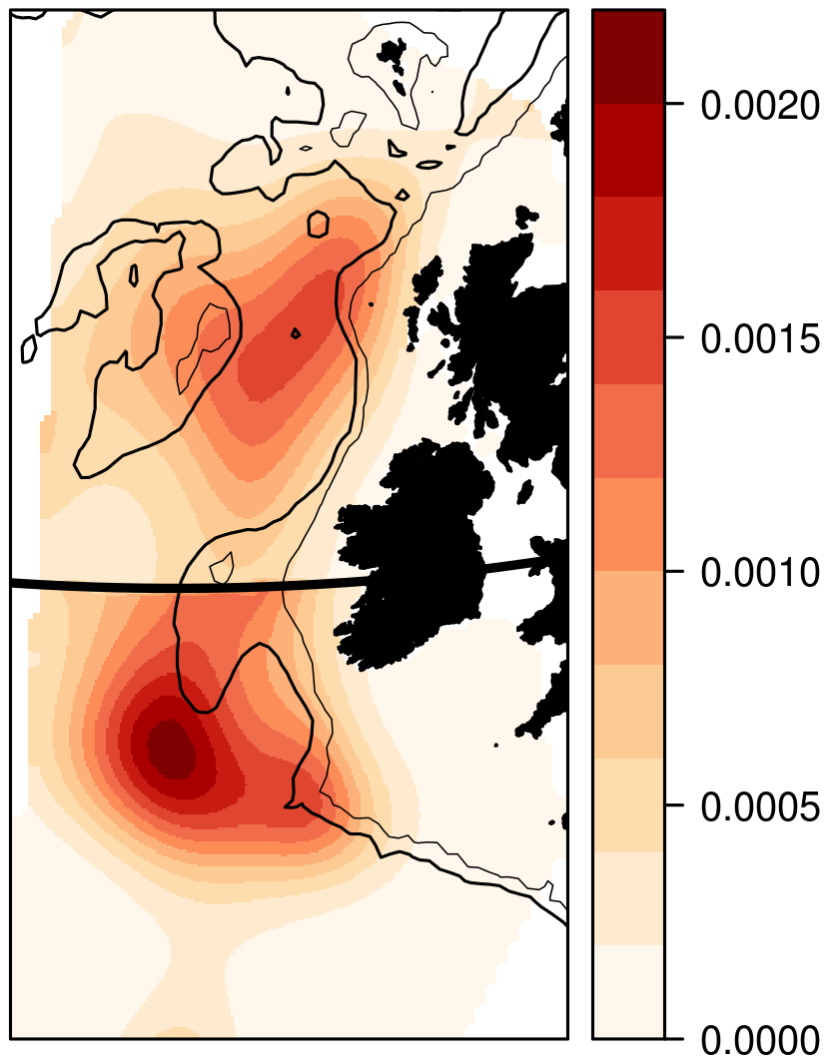
Spatial larval-presence probability distribution. Results predicted from Model 10 (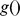  =  east * north * doy + s(year)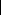 comp) are plotted as a probability density function for each population (*i.e.* the spatial integral over the domain of each of the two populations is 1). The black horizontal line indicates the location of the arbitrary division between a northern and southern population at 53 °N. Note abundances cannot be compared between the domains, as each domain is normalised to give an integral of 1. Isobaths are draw at 200 m (thin line) and 1000 m (thicker line) depths for reference. Map projection is UTM Zone 28.

The spatial patterns apparent in the simpler one-component models, are also clearly apparent in the best-fitting two-component model, Model 10. There appear to be two main centres of larval density (Figure 6). The first is in the Rockall Trough in the deep water off the continental shelf-edge to the north-west of Ireland and west of the Outer Hebrides. A second high-density region is centred south of the Porcupine Bank and south-west of Ireland, offshore from the Porcupine Seabight. Importantly, there appears to be a clear minimum between these two regions, hinting at their independence (Figure 6).

**Figure 7:**
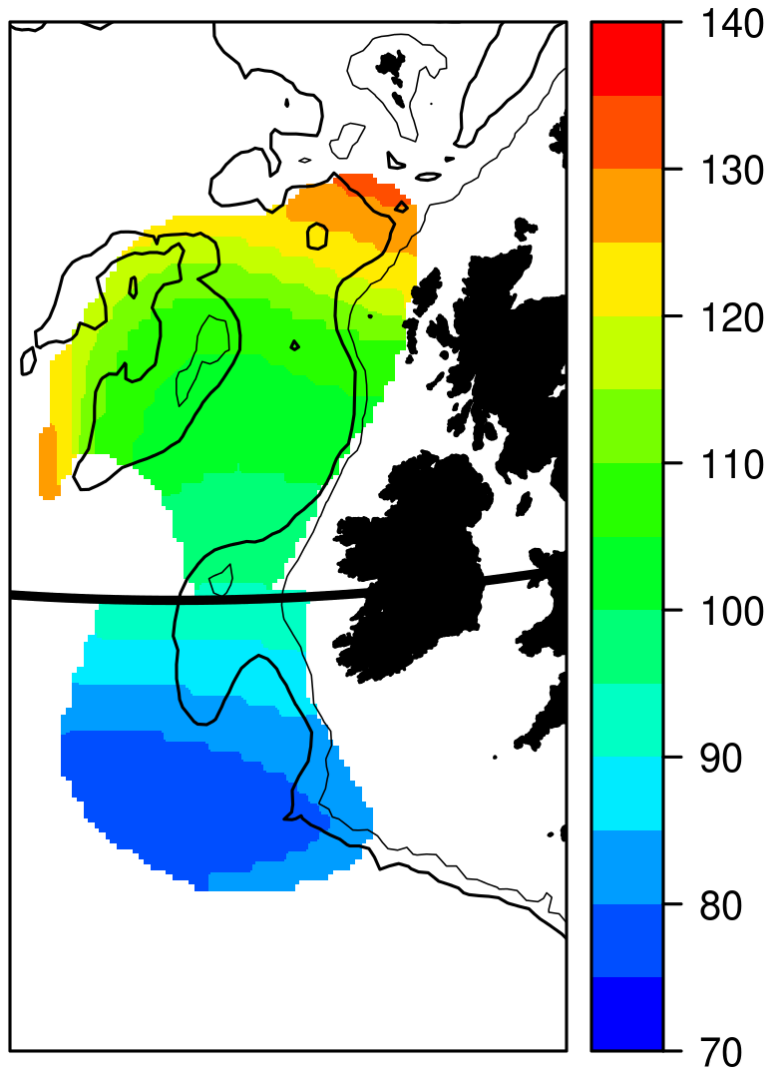
Timing of peak probability of occurrence. Results predicted from Model 10 (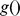  =  east * north * doy + s(year)
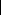
comp). The day of year (colour scale) when the local maximum in probability of larval presence occurs is plotted as a function of space. The black horizontal line indicates the location of the arbitrary division between a northern and southern population (at 53 °N). The spatial distribution in Figure 6 is used to mask the output so that only the core 75% of the larval distribution in each region is plotted: regions where there are few larvae, and the estimated timing of spawning is therefore imprecise, are thus omitted. Isobaths are draw at 200 m (thin line) and 1000 m (thicker line) depths for reference. Map projection is UTM Zone 28.

The two centres also clearly exhibit different distributions in the timing of spawning. The timing of the local maximum in larval density (Figure 7) is strongly dependent on space, exhibiting a systematic increase from the south to the north. The core of the two centres appear to differ substantially in the timing of maximum larval-density.

**Figure 8:**
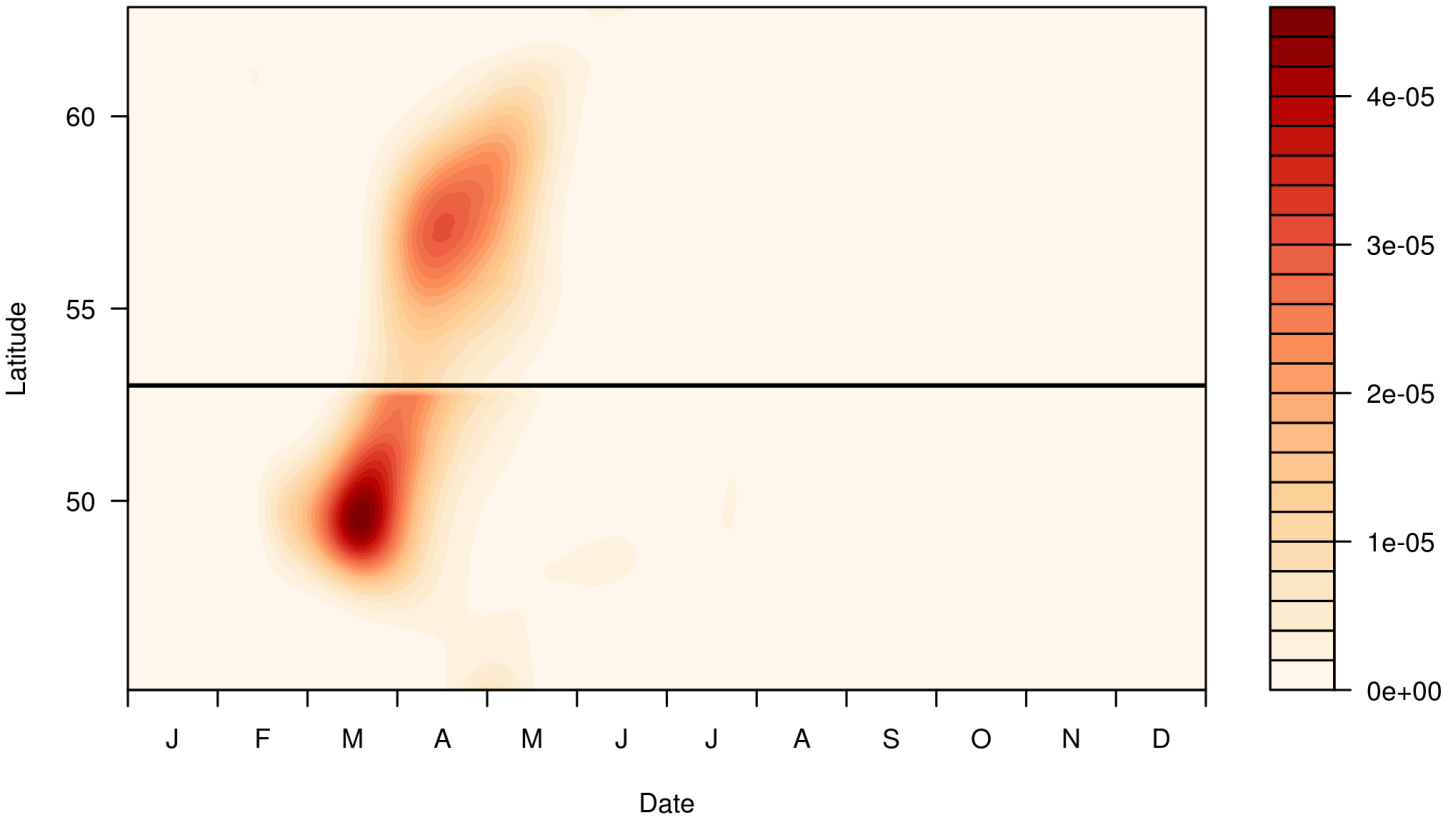
Zonally integrated larval-presence probability distribution. Results from Model 10 (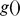  =  east * north * doy + s(year)
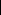
comp), plotting the probability distribution of larval-presence as a function of latitude and day of year. The probability of larval-presence is expressed as a density function for each population (*i.e.* the integral over each of the two populations is 1). The black horizontal line indicates the location of a hypothesised division between a northern and southern spawning population (at 53 °N). Note that because this model allows the relative abundances of the two populations to vary from year to year, abundances cannot be compared between the domains. The projected UTM coordinates used in the fitted model have been reprojected back to longitude here for ease of interpretation.

The zonal dependence of the larval temporal distribution is clearly apparent when the meridional dimension is integrated out (Figure 8). The temporal distribution of larval from the southern component appears to lead the northern component by at least 30–45 days. Furthermore, the temporal distribution of the northern component appears more protracted than that in the south, with appreciable larval densities into mid- and late-May.

**Figure 9:**
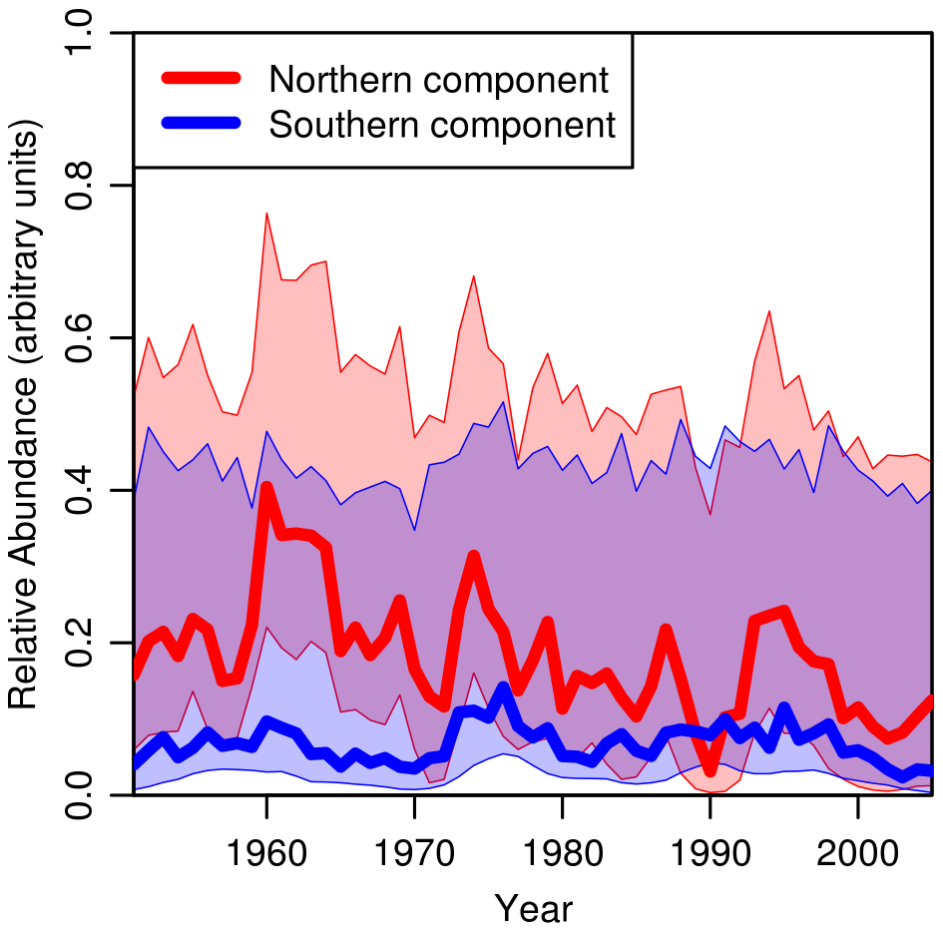
Annually integrated larval occurrence-probability. Results from Model 10 (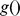  =  east * north * doy + s(year)
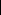
comp). The probability of observing larvae integrated over the spatial domain and day of year is a measure of larval abundance in that year and is plotted as a function of the year for the northern (red) and southern (blue) populations, with the associated 67% (*i.e.* corresponding to 1 standard deviation) confidence intervals. The units of larval abundance plotted here are arbitrary but scale linearly.

The overall abundance of the two components also appear to show different interannual dynamics (Figure 9). However, the confidence intervals about the median estimate are large, a result that is unsurprising given the poor sampling coverage in some years. The high uncertainty means that it is not appropriate to draw inference about the trends, nor to make comparisons with, for example, the spawning stock biomass from the stock assessment. Nevertheless, incorporating different interannual dynamics for the two components (from Model 6 to Model 10) resulted in a greatly improved fit to the data *i.e.* the abundance trends in each component are statistically different. Furthermore, although we have not tested it explicitly, the results clearly suggest that the southern component typically has an integrated abundance that is smaller, on average, than the northern component.

Finally, the day-night (*DN*) factor for the best fitting model, Model 10, was 0.17 (with a 95% confidence interval of [0.05,0.35]). All models showed comparable values for this factor. When translated into actual catchability, this results suggests that the CPR is marginally more effective at capturing larvae during the day than it is during the night. Such a result is not consistent with active avoidance of the sampler, where one would expect a reduced probability of capturing larvae during daylight hours. Instead, the result suggests diel vertical migration, where the larvae migrate close to the surface during the day and are therefore more readily captured by the CPR sampler.

## Discussion

### Reliability of the CPR data

In this study we infer the spatial, seasonal and interannual variability in the spawning of blue whiting from the presence and absence of blue whiting larvae in Continuous Plankton Recorder (CPR) samples. We argue that this is a valid proxy for the distribution of blue whiting spawning. Blue whiting spawn at depths of between 300 m and 600 m and, once hatched, rise to surface waters over the course of the first two-three weeks of life: larval length upon reaching these waters is 2–5 mm [45]. These field observations agree with the larval length distributions of blue whiting in the CPR reported by [25], who found all but a small minority of the larvae (approximately 5–10%) to be smaller than 6 mm. For contrast, while the length-at-metamorphosis of blue whiting is unknown [7], 15 mm larvae have been observed in other studies (*e.g.* [46], [47]) and there is a single report of a 42 mm larvae [48]. Similarly, Coombs *et al.* [49] performed detailed studies of blue whiting egg and larval development in the laboratory and demonstrated that yolk-sack absorption is complete after two weeks, at which point the larvae are approximately 5 mm in length. The blue whiting larvae in the CPR are highly-likely to be early-larvae, and their abundance therefore is likely to reflect the distribution of the adults that spawned them.

The choice of a presence/absence model, rather than a fully-developed abundance model, could potentially provide problems in interpretation. However, we note that single larvae are the most frequently observed class, and thus will have the strongest influence on an abundance-based model anyway. A reliable abundance model may also be difficult to develop due to the likely patchiness (and therefore overdispersion and zero-inflation) in the spatial and temporal distribution, and could easily be dominated by a few large catches. Nevertheless, future work should examine the use of the abundance categories in more detail.

This work provides another example of the utility of the CPR for investigating the characteristics of fish populations [33], [50], [51]. The study of the spawning distribution of this species in this region using fisheries surveys is made extremely difficult by the large areas over which blue whiting spawn: more than 1500 km north-south and 500 km east-west. In spite of the small flotilla of vessels typically used to cover this region, developing a synoptic picture of the distribution of this fish is challenging: multiple snapshots, enabling the dynamics of the spawning process to be tracked throughout the season, are simply not feasible. On the other hand, at least prior to the 1980s, the CPR provides observations with broad spatial and temporal coverage. Furthermore, the long time-series and consistency of the method allow insights into both the history and population structure of this species that would not otherwise be possible.

This study, however, also highlights some of the limitations of CPR data. The irregular, and varying sampling pattern, with many gaps in coverage, the low frequency of larval occurrence, and the use of categorical abundances make the analysis of this data challenging. Nevertheless, the development of modern statistical tools, combined with ready access to powerful computers, have opened up many new possibilities. In particular, the development of Generalised Additive Models, and their packaging in a user-friendly form (*e.g.* [36]) allow for non-Gaussian responses (presence-absence) to be modelled with complex predictors (*e.g.* the eastings-northings-day-of-year tensor-product smoothers employed here). Such tools were not available even a decade ago, and offer great potential for the future use of CPR data.

However, although these technical challenges can be solved, the most important limitation of the CPR for this study, the reduction in the sampling coverage in the Rockall region during recent times, cannot. Routes through the Rockall region have been reduced in frequency since the 1980s, and have been virtually eliminated since the 2000s (*e.g.* Figure S1), at least during the spring spawning-period of this species. These changes are unfortunate as these are the time periods that coincide with the modern fishery, the advent of scientific surveys, and the interesting scientific questions concerning population dynamics and the influence of the physical environment on this stock [4], [21], [32]. The current CPR spatial distribution is inadequate for monitoring this stock in this region: the reintroduction of regular haul lines through this area would be of great benefit to both the blue whiting community and all pelagic science performed in this region.

The reduced sampling also prevents extraction of useful measures of interannual variability from this data. Other studies have shown that the spatial distribution of blue whiting varies from year to year in concert with the sub-polar gyre [21], [32]. Unfortunately, the poor coverage means that it is probably not possible to study these processes based on CPR observations, at least during the post-1990s changes described elsewhere. Similarly, the poor precision in the modelled abundance estimates means that direct comparisons against the stock assessment, for example, are not practical. Analyses of interannual variability in both abundance and spatial distribution prior to 1980, where the spatial coverage is much greater, may be feasible, but are made more challenging by the lack of other data during this time. Instead, focus should be placed here upon the spatial (Figure 6) and seasonal (Figure 8) distributions of larvae. Disregarding the interannual processes, these results therefore become a form of climatological distribution averaged over the entire 55-year period for which CPR observations are available.

One potential weak point of our analysis is the post-hoc modification of the model ensemble to include two-component models, which represents a form of data-dredging [31]. However, this modification has a solid and independent scientific basis to support it and two-component models could therefore have been included in the original ensemble. Furthermore, we have chosen to be transparent about where this step fits in the modelling process, and we present results from both the original and expanded ensembles. Importantly, we note that the separation of the spawning-grounds in both time and space is clear in models from both the original and expanded ensembles. Thus, although a small amount of data-dredging has occurred in this work, we feel it is to an acceptable degree and do not believe that the validity of our results are unduly affected by it.

### A Resolution to the Paradox?

Our results suggest the presence of two unique spawning components. There is a clear separation between the two spawning centres, with a minimum in spawning activity occurring between 52 and 54 °N. Furthermore, we have also shown a difference in the timing of spawning of around a month between the two populations: in particular, spawning on the southern spawning ground appears to be nearly finished before it starts on the northern ground (*c.f.* Figure 8). Finally, we have shown a difference in the interannual abundances of these two components: although there is a large amount of noise in the interannual abundance estimates, a model (Model 10) with different interannual variations between the components is statistically superior to one (Model 6) assuming a common trend (*c.f.* Table 1).

Furthermore, the spatial separation into two spawning components closely mirrors the results obtained elsewhere, particularly from particle tracking studies. Bartsch *et al.* [16] suggested a separation between the populations at around 53/54 °N, whilst based on a different oceanographic model Svendsen *et al.* [17] and Skogen *et al.* [18] suggested a similar line at 54.5 °N. Here we chose a separation line at 53 °N, but the choice is essentially arbitrary and there appears to be a clear region of zero or minimal spawning between the components that also encompasses the aforementioned separation lines. Our direct observations of blue whiting larval distributions are therefore in line with these results.

Most importantly, our results suggest a resolution to the blue whiting population paradox. The crux of the problem is the supposed lack of evidence supporting the separation of the two hypothesised populations on the spawning grounds. However, we propose that this picture is simply an artefact of survey design. For example, the most recent (2013) survey took place over two weeks at the very end of March and the beginning of April and stretched from 53 °N to 62 °N (Figure 2) [52]. Such a survey will not capture spawning in the southern population for two reasons. Firstly, it occurs too late: the abundance of larvae in the southern population is essentially zero by the end of March (*c.f.* Figure 8), and therefore spawning, occurring approximately two weeks earlier than the larvae that we observed, peaked at least one month prior. Noting the highly migratory nature of blue whiting, it is not unreasonable to expect that the fish may have left the southern spawning grounds by late March.

Secondly, the survey does not extend sufficiently far south. The current survey design stops at Porcupine Bank (53 °N: Figure 2), whereas the southern population spawns offshore from the Porcupine Seabight, between 48 and 52 °N (Figure 6). Such an omission is not unique to modern times: a review of all acoustic surveys [32] shows regular coverage of Porcupine Bank, but not further south into the seabight where we suggest the southern population spawns.

We therefore conclude that the blue whiting population paradox is simply an observational artefact. While the distribution of the spawning products is clearly and cleanly separated in space and time, the acoustic observations of adult fish are not capable of resolving the southern population due to their restricted temporal and spatial coverage. Confusion therefore arises because the observations are only capable of capturing the northern population, rather than both populations, creating the (false) appearance of a continuous distribution on the spawning grounds.

With the insight afforded by these new results, an important inconsistency in the literature becomes apparent. Many authors have previously considered the Porcupine Bank to be the spawning ground of the southern population and designed their studies accordingly: however, these results suggest that the north-side of the Porcupine Bank should be considered as northern “territory” (*e.g.* Figure 6). This revelation suggests that the interpretation of many existing studies need to be reconsidered. For example, the results of a microsatellite genetic study on blue whiting population structure [12] lumped the north-side of the Porcupine Bank together with the Outer Hebrides and Rockall Plateau, whilst samples taken from the Porcupine Seabight were genetically distinct. To a researcher working under the (previous) assumption that the Porcupine Bank is the “southern” component, these results are confusing. However, when combined with the results presented here, where Porcupine Bank is part of the northern population, they are consistent. Similar reinterpretations occur when re-examining the otolith juvenile growth [13] and shape [14] studies. Furthermore, observational studies reporting spawning fish off the Porcupine Seabight [53], which made little sense in the previous conceptual model, now give both meaning and lend their support. There is therefore a need for a comprehensive re-examination of the published literature on this topic: however, that is clearly beyond the scope of this work.

Nevertheless, and other studies not withstanding, there is now clear evidence that the North Atlantic blue whiting population should be considered as two independent stocks. Studies based on both genetics [12] and otoliths [13], [14] support this separation, while circulation studies [16]–[18] provide a physical mechanism that maintains the separation between the larvae spawned in these two locations. In this study, we have shown a clear physical separation between the two populations, and that there is at least a month difference in the timing of peak spawning. Furthermore, the interannual variations in the abundances of each population are also statistically different. With the lack of structure in the adult observations now explained as an observational artefact, the case for two-populations already appears irresistibly strong.

The current management paradigm, however, is based on a single stock approach and is likely to be so for some time to come. In contrast to early assessments (*e.g.* [19]), little attention is paid to quantifying the southern population and there is therefore a risk of inadvertently fishing it to collapse. Studies in other small pelagic species (*e.g.* herring, *Clupea harengus*) suggest that maintaining stock/population diversity provides resilience against both natural and anthropogenic stresses and helps maintain productivity [54]–[56]. However, even in the absence of separating these two populations into unique management units, improvements in the monitoring of these populations are possible. The most obvious is the extension of the spawning acoustic survey both in space and time to cover the spawning of the southern population. Secondly, the re-establishment of CPR haul lines through the Rockall region would allow direct comparison with modern observations, and therefore aid the interpretation of the historical CPR observations. Such changes should be considered as critical steps towards the precautionary management of blue whiting in the North Atlantic.

## Supporting Information

Figure S1 **Annual spring distribution of CPR samples.** Samples checked for fish larvae obtained from the CPR. Grey points are locations where CPR samples have been checked for fish larvae. Red circles are where these samples were found to contain blue whiting. As blue whiting larvae are predominately captured in the first half of the year, only observations from January to June (inclusive) are plotted here. Map projection is UTM Zone 28.
(TIFF)

Figure S2
**Spatial larval-presence probability distribution.** Results from Model 6 (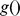  =  east * north * doy + s(year)), plotted as a probability density function (*i.e.* the spatial integral over the domain is 1). Isobaths are draw at 200 m (thin line) and 1000 m (thicker line) depths for reference. Map projection is UTM Zone 28.(TIFF)

Figure S3
**Zonally integrated probability distribution.** Results from Model 6 (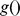  =  east * north * doy + s(year)), plotting larval occurrence probability as a function of latitude and day of year. The probability of larval-occurrence is expressed as a probability density function (*i.e.* the integral over the domain is 1). The UTM coordinates used in the fitted model have been reprojected back to longitude for ease of interpretation.(TIFF)
