# Supplementary figures and images for: A Resolution to the Blue Whiting (Micromesistius poutassou) Population Paradox?
Source: PLoS One. 2014 Sep 3;9(9):e106237. doi: 10.1371/journal.pone.0106237 (PMC4153562; doi:10.1371/journal.pone.0106237)

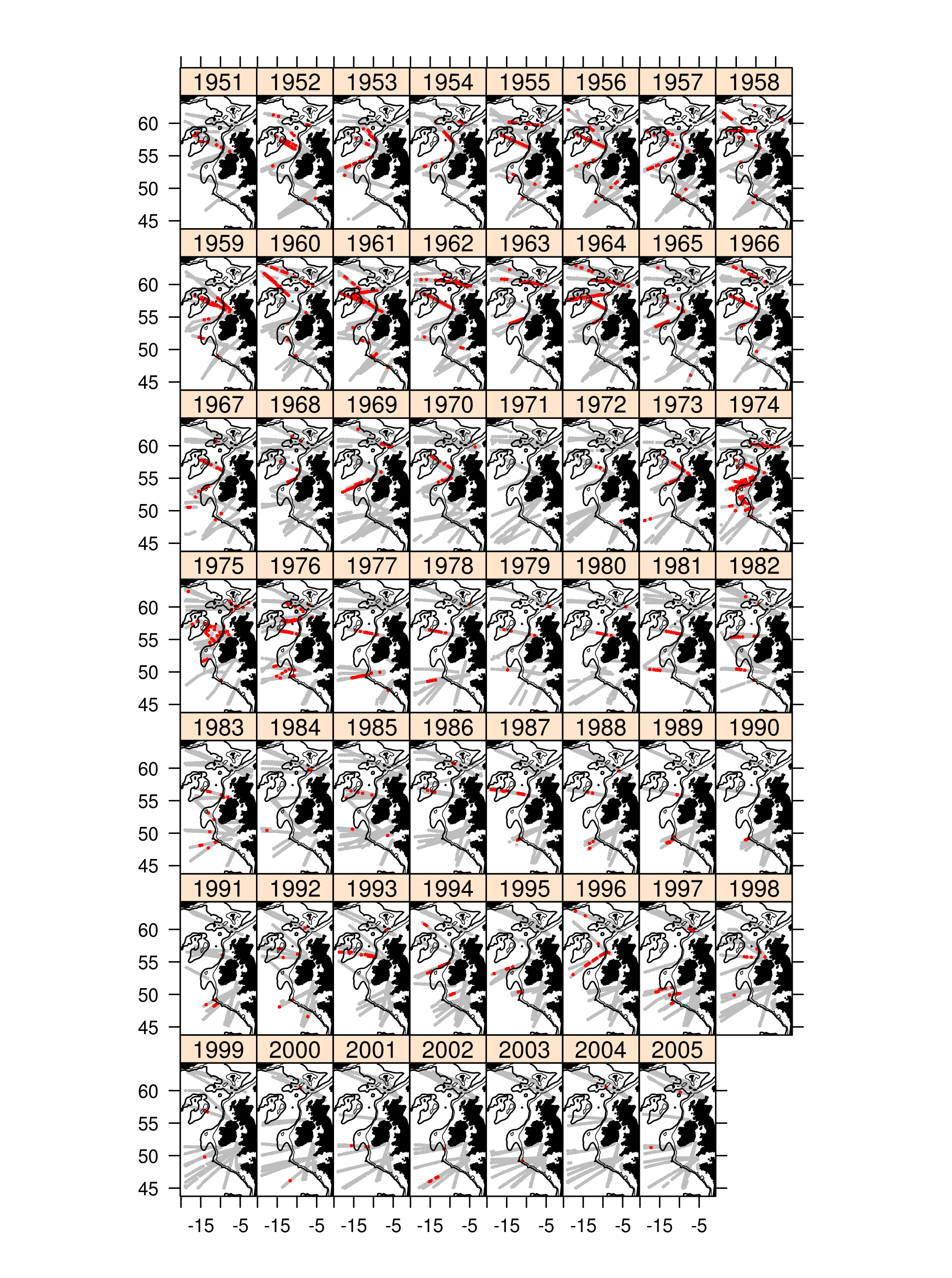

Supplement: Figure S1 — Annual spring distribution of CPR samples. Samples checked for fish larvae obtained from the CPR. Grey points are locations where CPR samples have been checked for fish larvae. Red circles are where these samples were found to contain blue whiting. As blue whiting larvae are predominately captured in the first half of the year, only observations from January to June (inclusive) are plotted here. Map projection is UTM Zone 28. (TIFF) [file pone.0106237.s001.tiff]

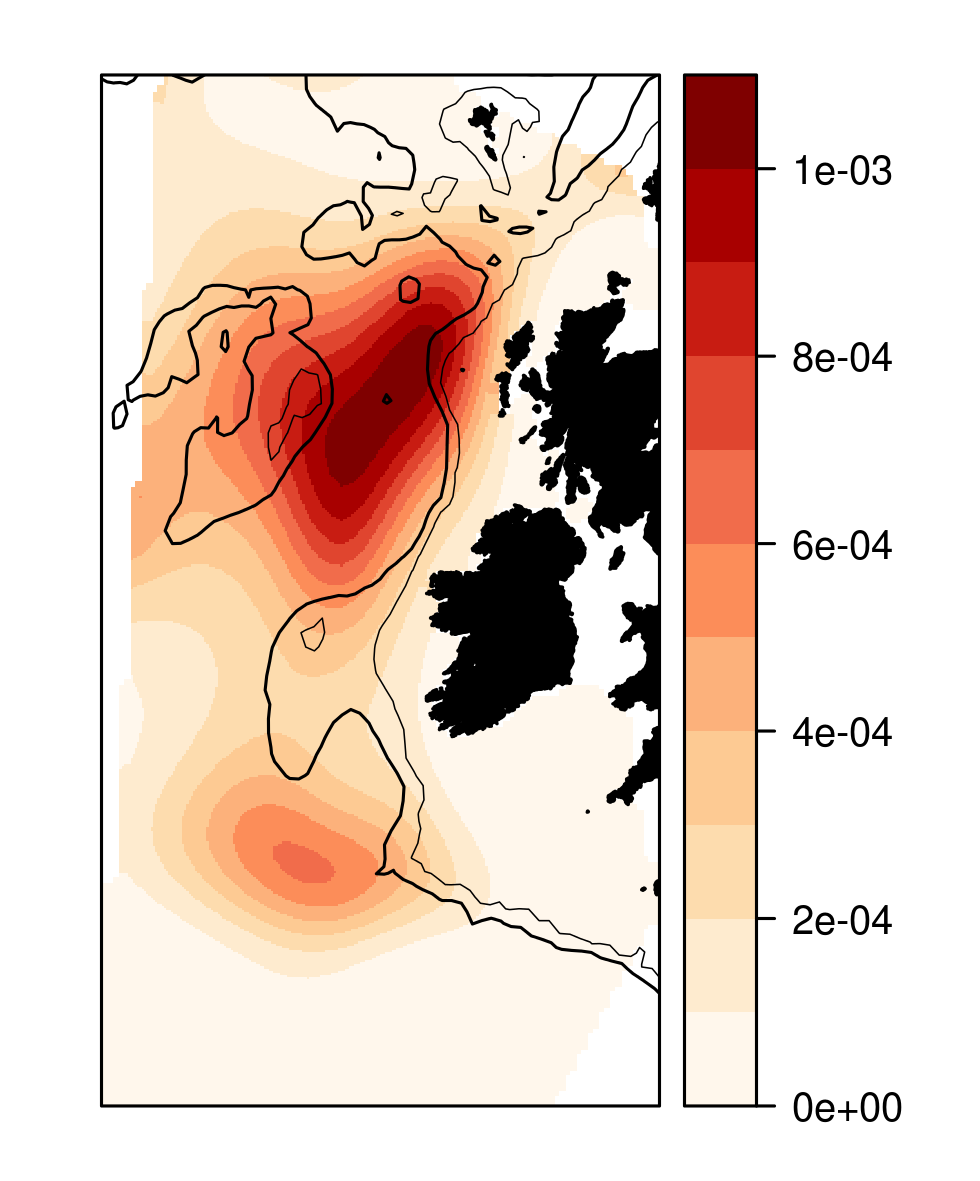

Supplement: Figure S2 — Spatial larval-presence probability distribution. Results from Model 6 ( = east * north * doy + s(year)), plotted as a probability density function (i.e. the spatial integral over the domain is 1). Isobaths are draw at 200 m (thin line) and 1000 m (thicker line) depths for reference. Map projection is UTM Zone 28. (TIFF) [file pone.0106237.s002.tiff]

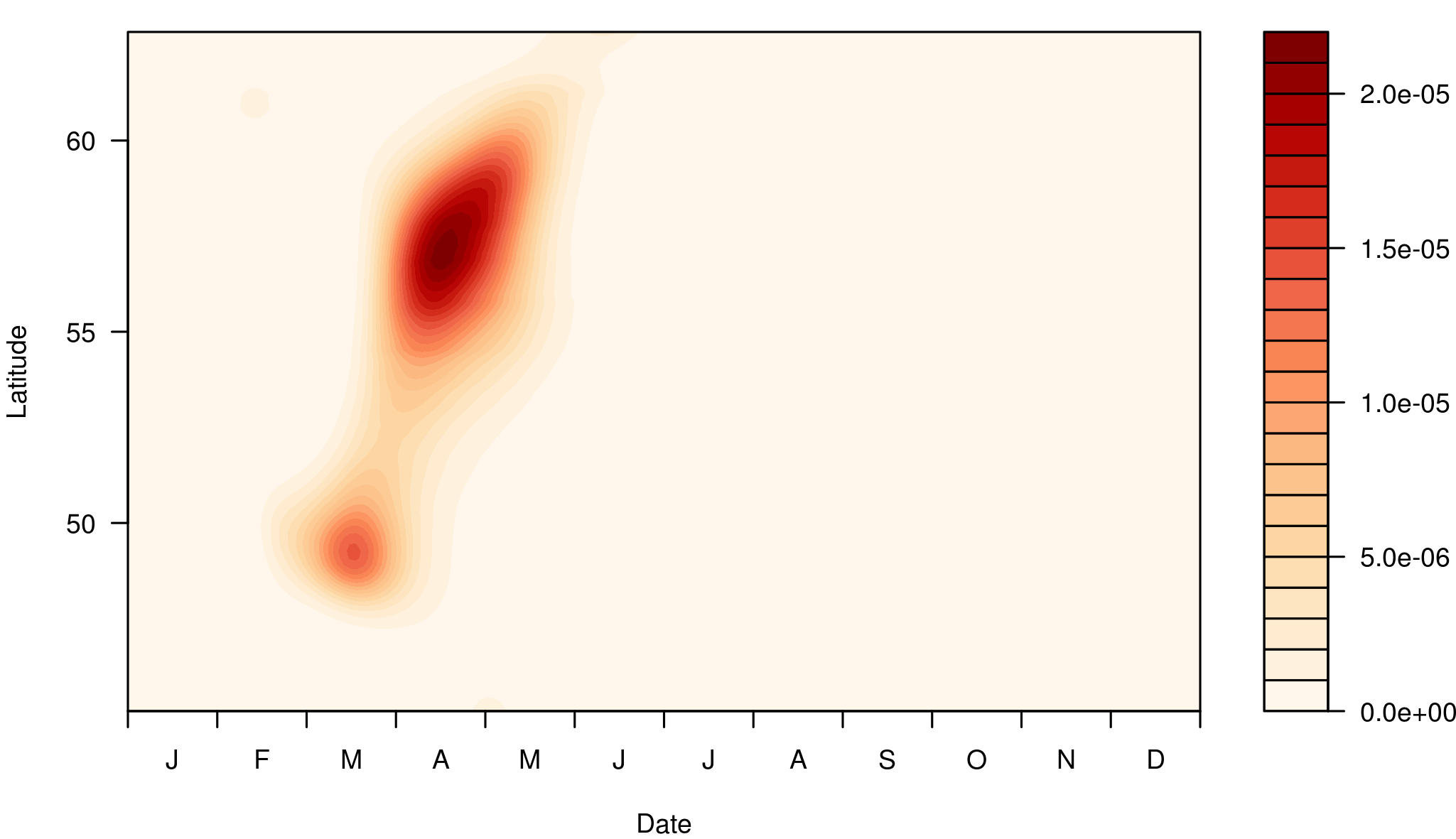

Supplement: Figure S3 — Zonally integrated probability distribution. Results from Model 6 ( = east * north * doy + s(year)), plotting larval occurrence probability as a function of latitude and day of year. The probability of larval-occurrence is expressed as a probability density function (i.e. the integral over the domain is 1). The UTM coordinates used in the fitted model have been reprojected back to longitude for ease of interpretation. (TIFF) [file pone.0106237.s003.tiff]
